# Fructose and Trehalose Selectively Enhance In Vitro Sporulation of *Paenibacillus larvae* ERIC I and ERIC II Strains

**DOI:** 10.3390/microorganisms9020225

**Published:** 2021-01-22

**Authors:** Maroš Laho, Mária Šedivá, Juraj Majtán, Jaroslav Klaudiny

**Affiliations:** 1 Institute of Chemistry, Slovak Academy of Sciences, Dúbravská cesta 9, 84538 Bratislava, Slovakia; maros.laho@savba.sk (M.L.); maria.sediva@savba.sk (M.Š.); 2 Institute of Molecular Biology, Slovak Academy of Sciences, Dúbravská cesta 21, 84551 Bratislava, Slovakia; juraj.majtan@savba.sk

**Keywords:** honeybee, American foulbrood, *P. larvae*, saccharide, bacterial spores, spore germination, spore preparation, ERIC genotype, royal jelly, honey

## Abstract

*Paenibacillus larvae* is a Gram-positive bacterium, the spores of which are the causative agent of the most destructive brood disease of honeybees, American foulbrood (AFB). Obtaining viable spores of pathogen strains is requisite for different studies concerning AFB. The aim of this work was to investigate the effects of five saccharides that may naturally occur in higher amounts in bee larvae on in vitro sporulation of *P. larvae*. The effect of individual saccharides at different concentrations on spore yields of *P. larvae* strains of epidemiologically important ERIC genotypes was examined in Columbia sheep blood agar (CSA) and MYPGP agar media. It was found that fructose in ERIC I and trehalose in ERIC II strains at concentrations in the range of 0.5–2% represent new sporulation factors that significantly enhanced the yields of viable spores in both media, mostly in a concentration-dependent manner. The enhancements in spore yield were mainly caused by improvements of the germination ability of the spores produced. Glucose, maltose and sucrose at 1% or 0.5% concentrations also supported sporulation but to a lower extent and not in all strains and media. Based on the knowledge gained, a novel procedure was proposed for the preparation of viable *P. larvae* spores with supposed improved quality for AFB research.

## 1. Introduction

American foulbrood (AFB) causes large annual worldwide financial losses to beekeepers and farmers dependent on crop pollination. The primary event in disease development in a colony is the infection of young larvae with *Paenibacillus larvae* spores, which contaminate larval food [1,2,3]. Ingested spores germinate in the larval midgut where the infection continues by massive proliferation of vegetative cells [4] associated with the production of substances that help bacteria to penetrate through the peritrophic matrix and midgut epithelium into the hemocoel (reviewed in [5,6]). Invasion of the hemocoel with pathogen cells leads to the death of larvae, their degradation to a ropy mass and sporulation of the pathogen; eventually, billions of spores occur in dried scales adhering to cell walls. The spores are transmitted by worker bees to other larvae and escalation of the infection in the colony leads to its collapse [3,7].

The disease is highly contagious. Spores are spread among colonies by bees and also by beekeepers [8,9,10]. When various preventive measures [8,11] fail and colonies become clinically infested they can either be cured with antibiotics (allowed in some countries, including the United States and Canada) [12,13,14] or are destroyed by burning, together with the associated hive materials (EU and most other countries) [3,15]. The use of antibiotics may be problematic because it can be connected with honey contamination [16,17] and their efficacy can impact the development of resistant strains of the pathogen [18,19,20].

Honeybee colonies differ in resistance to AFB [3,8,21,22]. Their resistance lies in immune responses acting in individual larvae [23,24,25,26,27] and social defence mechanisms mediated by the workers [28,29,30]. It is also assumed that the resistance of individual larvae can be affected by the larval food, which contains honeybee-derived antibacterial substances, some of them with proven in vitro efficacy against *P. larvae* (reviewed in [31]). It is probable that the levels of most of the defence mechanisms in individual larvae and workers are determined genetically and therefore can be influenced by breeding. The breeding of resistant lines represents another way (besides antibiotic treatment and colony burnings) to control AFB and its sense has been shown by breeding colonies with hygienic behaviour [30,32,33]. Moreover, the possible use of different natural substances and probiotic bacteria for the prevention and control of AFB has been widely studied (reviewed in [34,35]), as well as employing bacteriophages for AFB prevention or treatment (reviewed in [36]).

To study the various issues concerning infection with AFB, viable spores are needed. They are used in investigations of pathobiological and immune processes acting in *P. larvae* infection in worker or drone larvae in one or more colonies [37,38,39,40,41,42,43,44,45] and to determine the pathogenicity of different *P. larvae* strains/isolates for individual larvae and whole colonies. Most of such studies have been done with exposure bioassays based on infections of larvae reared in vitro with spores. In this way, it was revealed that *P. larvae* strains with the ERIC I genotype (ERIC—Enterobacterial Repetitive Intergenic Consensus sequences) are less virulent for individual larvae than the more virulent ERIC II–V strains (larvae are killed slowly by ERIC I, with medium speed by ERIC II and fast by ERIC III–V strains) [6,39,46]; in connection with the hygienic behaviour of bees, this leads to high-level spore production and faster collapse of colonies infected with ERIC I than with ERIC II strains and likely caused the extinction of the ERIC III and ERIC IV strains from the environment [41]. Moreover, the spores are required for evaluation of the anti-*P. larvae* activity of various substances and bacteriophages by means of bioassays [47,48,49,50], as well as for examination of the antibacterial efficacy of substances on germinating spores with in vitro assays [31,48].

Viable spores can be prepared either from AFB scales [47,51] or by in vitro sporulation methods. Several procedures have been developed that achieve the effective sporulation of many *P. larvae* strains. These procedures employ different solid agar media (MYPGP, CSA and TSA—tryptic soy agar) or liquid medium (TMYPG) for cell growth and sporulation and can differ in some cultivation conditions, such as certain temperatures, sporulation times, and incubation in an aerobic or partially anaerobic atmosphere [39,52,53,54,55,56].

We have recently used the standard spore preparation method employing MYPGP agar plates (recommended for AFB research by [54]) for in vitro determination of the antibacterial efficacy of *trans*-10-hydroxy-2-decenoic acid (10-HDA) on germinating *P. larvae*. In the process, we were met with poor sporulation of some our experimental strains [31]. We solved that problem and prepared sufficient spores for our tests with reiterated spore preparations performed with prolonged sporulation. Nevertheless, we realized that, for the intended in vivo infection experiments of larvae with bioassays, we need a procedure allowing us to prepare the required larger amounts of spores more effectively. We decided not to seek a better-functioning procedure among those already published but to investigate our idea that some saccharides that may naturally occur in higher amounts in food and bodies of honeybee larvae could help to improve the sporulation of problematic strains.

In this work, we studied the effect of five saccharides added individually at higher concentrations to MYPGP and CSA plates on the spore yields of three ERIC I and three ERIC II *P. larvae* strains. We searched for the concentrations at which the most effective saccharides (fructose and trehalose) are most capable of affecting the yields of viable spores of all strains tested. The fundamentals of improved sporulation of strains with higher fructose or trehalose concentrations in media were also investigated. Various matters concerning sporulation of *P. larvae* strains at higher saccharide concentrations are discussed as well as the significance of our findings for the preparation of spores for AFB research. A novel procedure for preparing viable *P. larvae* spores is suggested and its more variant character is described in detail.

## 2. Materials and Methods

### 2.1. P. larvae Strains

Four reference strains of *P. larvae*, three possessing an ERIC I genotype—LMG 9820 (other designations DSM 7030, ATCC 9545, NRRL B-2605), CCUG 28515 and CCM 4483—and one having an ERIC II genotype—CCM 4486—were purchased from different culture collections. Two field isolates of *P. larvae* with an ERIC II genotype—PL 2/11 and PL 4/11—were obtained from AFB-positive hives (from wax debris) in Slovakia (2011). The ERIC genotypes of the strains and isolates were verified in our previous work [31].

### 2.2. Saccharides

D-Fructose (≥99%), D-glucose (≥99%), sucrose (≥99%) and maltose (≥98%) were purchased from Sigma-Aldrich, Europe; trehalose (>98%) was purchased from TCI Europe N.V.

### 2.3. Standard Sporulation Protocol

A slightly modified version of the standard protocol described by de Graaf et al. [54] was used as the reference procedure for preparation of spores. Overnight cultures of *P. larvae* strains were prepared on a rotary shaker at 35 °C in MYPGP medium (pH 7.2). From them, about 1000 cell units (individual units contained one or more chained vegetative cells—counted in a Bürker chamber) were inoculated on MYPGP agar plates (pH 7.2) or commercially available CSA plates (Columbia agar with sheep blood, OXOID, Code: PB0123). The compositions of the basal media were described by [54]. Plates containing 500–1500 growing colonies were used for further cultivation that was performed at 35 °C for 7 or 10 days. Grown colonies were removed from the surface of the agar plate by rinsing repeatedly three times with 5 mL of sterile, cold (4 °C) deionized water and colony rubbing with a glass cell spreader. The collected suspension was homogenized, and the bacterial mass washed four times by repetition of the vortexing and centrifugation (15,000 × *g*, 15 min, 4 °C) in 15 mL of sterile, cold deionized water in centrifuge tubes (15 mL; Labcon, Petaluma, CA, USA). The pelleted material was suspended in 2 mL of cold sterile deionized water and stored as spore stock culture in 2 mL Eppendorf tubes at 4 °C.

### 2.4. Sporulation on Agar Media Containing Different Saccharides

Stock solutions (20%) for every saccharide were prepared and sterilized by filtration through a 0.2 μm filter. MYPGP plates containing individual saccharides at the desired concentrations were prepared by adding the required volumes of stock solutions to an autoclaved medium before plating (after its cooling to 50 °C). CSA plates were enriched with saccharides by plating the stock solutions on the agar surfaces in the amounts needed to reach the required concentrations in the whole agar volume. The CSA plates were prepared one day before their use to ensure complete diffusion of saccharides within the agar. Sporulation and preparation of spore stock cultures from these plates were performed in the same way as described in the standard protocol.

### 2.5. Quantification of Spores and Determination of Spore Germination Rates

Prepared spore stock cultures were stored for 1–4 days in a refrigerator and then heated at 65 °C for 15 min to kill vegetative cells and immature heat-sensitive spores. The concentrations of viable (able to germinate) heat-resistant spores in cultures were determined as the number of colony-forming units (CFU)/mL after dilution of aliquots and their cultivation on MYPGP plates supplemented with germination factors (3 mM uric acid and 3 mM L-tyrosine; [55]) at 35 °C for 5 days. The concentrations of total spores (viable and inviable) in cultures were determined by microscopic spore counting in a dark field using a Bürker chamber at 400 × magnification. The determination was done in four to six cultures selected from each type of sample after determination of the viable spore concentrations in the cultures. The selected cultures contained viable spores in such concentrations that were closest to the average concentrations estimated in individual types of samples. Spore germination rates were determined according to the formula: concentration of viable spores/concentration of total spores × 100.

### 2.6. Data Analysis 

Statistical analyses were performed using GraphPad Prism 7 (GraphPad Software, LA Jolla, CA, USA) and IBM SPSS Statistics 23 (IBM Co., New York, NY, USA). Results are expressed as the mean ± standard error of the mean (SEM). The data acquired were first evaluated with regards to their normality of distribution by the Shapiro–Wilk test. Subsequently, the appropriate statistical method was selected and carried out, which was either a one-way analysis of variance (ANOVA) with a post-hoc Dunnett’s *t*-test or the Kruskal–Wallis nonparametric test with Dunn’s test post-hoc comparison. Data with *p* values smaller than 0.05 were considered statistically significant. In addition, the effect size (ε^2^) was calculated to evaluate the effect of different saccharides and interpreted according to [57]. 

## 3. Results

### 3.1. Effect of Cultivation Time on Sporulation in the Standard Protocol

**Figure 1:**
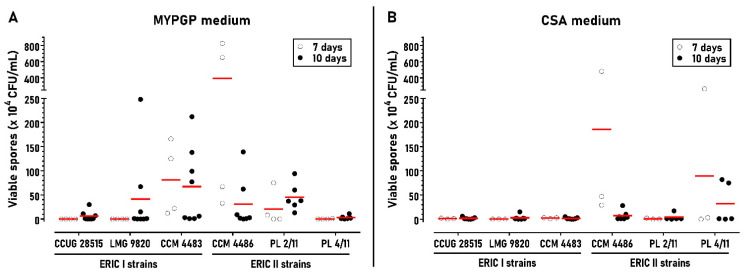
Effect of cultivation time on spore yields of *P. larvae* strains in the standard protocol. (**A**) Strains cultivated on MYPGP plates for 7 days (*n* = 4) or 10 days (*n* = 6–8). (**B**) Strains cultivated on CSA plates for 7 days (*n* = 3) or 10 days (*n* = 5–7). Red lines represent the means of the measured data.

Different sporulation protocols use different cultivation times for *P. larvae* cell growth and sporulation on agar plates: between 6 and 14 days when using MYPGP plates [52,54,58,59] and 10 days for CSA slants [39]. Our previous work suggested that the duration of cultivation may affect sporulation ([31] and unpublished results). Therefore, we firstly determined the spore yields of *P. larvae* strains on MYPGP and CSA plates for two cultivation times, 7 and 10 days. Three strains reached the highest average spore yields for 7-day incubation, either on MYPGP plates (CCM 4483) and CSA plates (PL 4/11) or on both plate types (CCM 4486). The other strains reached it after 10-day incubation. Generally, the strains sporulated better on MYPGP than CSA plates (Figure 1). Because the poorly sporulating strains sporulated better at 10 days on both agar media, we performed the subsequent sporulation tests with the strains using this cultivation time.

### 3.2. Effect of Different Individual Saccharides on Sporulation

**Figure 2:**
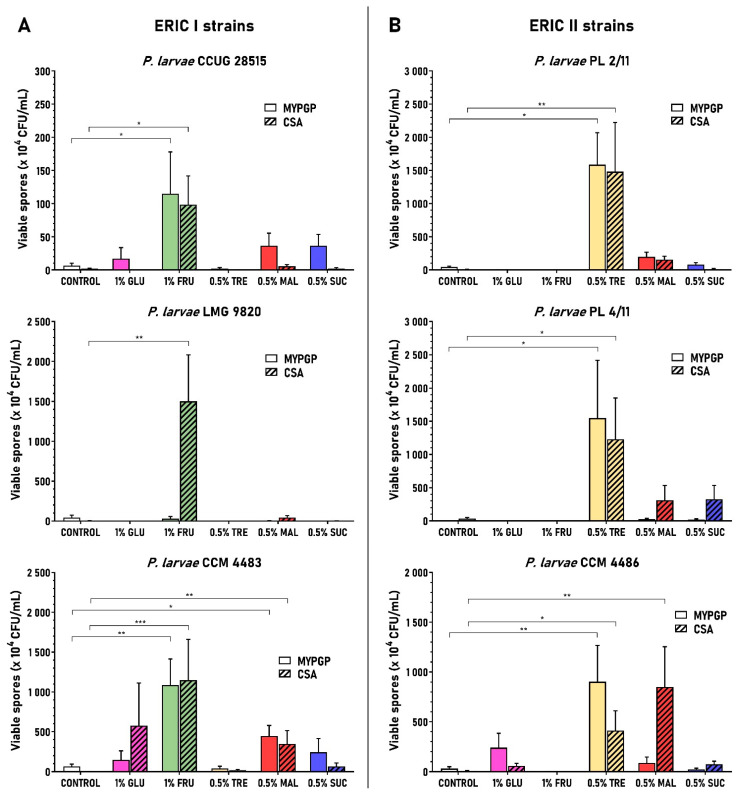
Effect of different saccharides on sporulation of the *P. larvae* ERIC I strains (**A**) and ERIC II strains (**B**). Strains were cultivated on MYPGP and CSA plates containing individual saccharides at 0.5% or 1% concentrations. The spore yields obtained with different saccharides are depicted in columns with different colours. Controls (without colour) represent yields obtained on plates not containing the saccharides. Spore yields are expressed as the mean ± SEM. Data showing statistical significance are marked (* *p* < 0.05, ** *p* < 0.01, *** *p* < 0.001). The number of replicates for the different spore cultivations were as follows: (**A**) MYPGP plates: controls *n* = 8, saccharide^+^ *n* = 6–7; CSA plates: controls *n* = 7, saccharide^+^ *n* = 5–6; (**B**) MYPGP plates: controls *n* = 6–7, saccharide^+^ *n* = 4–6; CSA plates: controls *n* = 5–7, saccharide^+^ *n* = 4–6.

The effects were examined at two concentrations of individual saccharides on MYPGP and CSA plates; the saccharides glucose and fructose, occurring in large quantities [60,61] in royal jelly and honey (a component of larval food), were added to the media at a 1% concentration; the other saccharides with a lower content in larval food [61,62,63,64] were added at 0.5% concentration. At these concentrations, the saccharides mostly affected sporulation positively in comparison with the control basal agar media. Statistical analysis showed that the saccharide supplements had a significant effect on viable spore yields (*p* < 0.05 to *p* < 0.0001), with an effect size (ε^2^) from 0.37 to 0.85, indicating their strong or very strong effects (except strain LMG 9820 cultivated on MYPGP plates). Bigger or smaller differences existed in the sporulation rates among the individual saccharides, different strains and media used. The biggest improvements in spore yield versus controls were obtained with fructose in the ERIC I genotype strains (*p* < 0.05 to *p* < 0.001) and trehalose in the ERIC II genotype strains (*p* < 0.05 to *p* < 0.01) in both agar media. The improvement concerned all strains poorly sporulating on the basal media as well as those sporulating better (Figure 2). The average spore yield versus controls within ERIC I strains increased 16.2–18.2-fold on MYPGP (except strain LMG 9820) and 57.8–717-fold on CSA plates. Within the ERIC II strains, there was a 29.2–483-fold increase on MYPGP and a 38.7–370-fold increase on CSA plates. Maltose and sucrose also improved the sporulation of most strains, mostly on both agar media, but the spore yields gained were lower than those for fructose and trehalose, with one exception (strain CCM 4486 on CSA plates). The average spore yield versus controls within strains of both ERIC genotypes increased 2.8–8.3-fold on MYPGP and 3.2–217-fold on CSA plates in the case of maltose, and 1.8–5.8-fold on MYPGP and 1.2–41.4-fold on CSA plates in the case of sucrose. Glucose improved sporulation in the smallest number of strains (in three strains); it increased the average spore yields 2.2–7.8-fold on MYPGP and 8.3–360-fold on CSA plates. Three saccharides—fructose, trehalose and glucose—also showed negative effects on average spore yields versus controls in several cases. Interestingly, they occurred in those strains and agar media where these saccharides did not support sporulation at all. In the case of fructose, it concerned all strains of the ERIC II genotypes; conversely, for trehalose, it concerned all strains with the ERIC I genotypes.

### 3.3. Concentration-Dependent Effect of Fructose on Sporulation of ERIC I Strains

**Figure 3:**
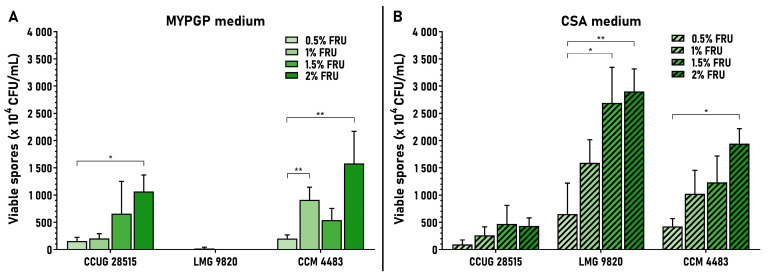
Effect of different fructose concentrations on sporulation of *P. larvae* ERIC I strains. Spore yields obtained on MYPGP or CSA plates containing 0.5–2% of fructose are depicted as the mean ± SEM. Statistical significance of data considering the yields obtained at 0.5% fructose as the control is shown (* *p* < 0.05, ** *p* < 0.01). Individual data sets represent results from 3–5 spore cultivation replicates at 0.5% and 1.5% fructose and 5–9 replicates at 1% and 2% fructose.

The effect was examined for fructose concentrations of 0.5% to 2% on MYPGP and CSA plates. Increasing the fructose concentration in this interval was associated with a continual increase of the average spore yield in almost all strains on both agar media. The exceptions were strain LMG 9820, where fructose did not affect sporulation on the MYPGP plates, and the two cases where continual growth of the yield was disturbed (CCM 4483 on MYPGP plates with 1.5% fructose, and CCUG 28515 on CSA plates with 2% fructose) (Figure 3). The improvements in average spore yield between 0.5% and 2% fructose were 6.9–7.8-fold on MYPGP and 4.4–4.6-fold on CSA plates. Comparing 2% versus 1% fructose (used primarily in tests), yield was improved 1.7–5.1-fold on MYPGP and 1.7–1.9-fold on CSA plates. The analyses performed revealed that 2% fructose had the biggest effect on the spore yields of ERIC I strains. Moreover, they showed that both types of agar media were useful for sporulation and preparation of spores from the ERIC I strains—the highest average spore yields were reached either on MYPGP plates (strain CCUG 28515) or CSA plates (LMG 9820 and CCM 4483 strains).

### 3.4. Concentration-Dependent Effect of Trehalose on Sporulation of ERIC II Strains

**Figure 4:**
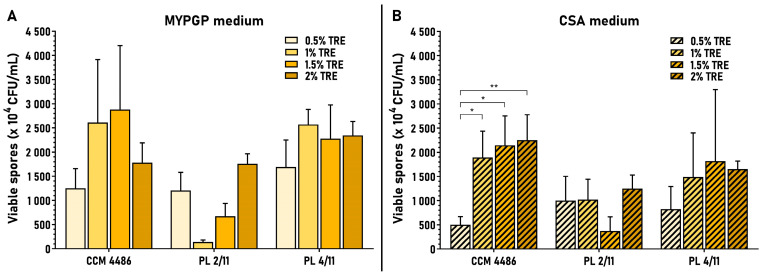
Effect of different trehalose concentrations on sporulation of *P. larvae* ERIC II strains. Spore yields obtained on MYPGP or CSA plates with 0.5–2% concentrations of trehalose are depicted as the mean ± SEM. Statistical significance of data considering the yields obtained at 0.5% trehalose as a control is shown (* *p* < 0.05, ** *p* < 0.01). Individual data sets represent results from 3–5 spore cultivation replicates at 1% and 1.5% trehalose and 6–9 replicates at 0.5% and 2% fructose.

The effect of trehalose on MYPGP and CSA plates was examined in the same concentration interval as for fructose, 0.5–2%. A sustained increase of average spore yields within these concentrations was observed only in one strain and medium (CCM 4486, CSA plates). Increases also occurred in other strains but not in the whole range of concentrations tested. In some cases, the highest spore yields were reached at lower concentrations, at 1% (PL 4/11, MYPGP plates) and 1.5% (CCM 4486, MYPGP plates and PL 4/11, CSA plates), or increases in spore yields were disturbed at certain concentrations: 1%, 1.5% and 1.5% (PL 2/11, MYPGP and CSA plates, respectively) (Figure 4). 

The greatest improvements in average spore yield achieved between 0.5% and different trehalose concentrations in individual strains were 1.5–2.3-fold on MYPGP plates and 1.2–4.5-fold on CSA plates. Evaluation of these results led us to the conclusion that 2% trehalose is commonly the most suitable saccharide for obtaining the highest or high spore yields in ERIC II strains. Moreover, we concluded that both types of agar demonstrated their usefulness for sporulation and preparation of spores from ERIC II strains—the highest average spore yields were reached either on MYPGP plates (PL 2/11 and PL 4/11 strains) or on CSA plates (strain CCM 4486) at 2% trehalose.

### 3.5. Effect of Fructose and Trehalose on Total Spore Production and Spore Germination

**Figure 5:**
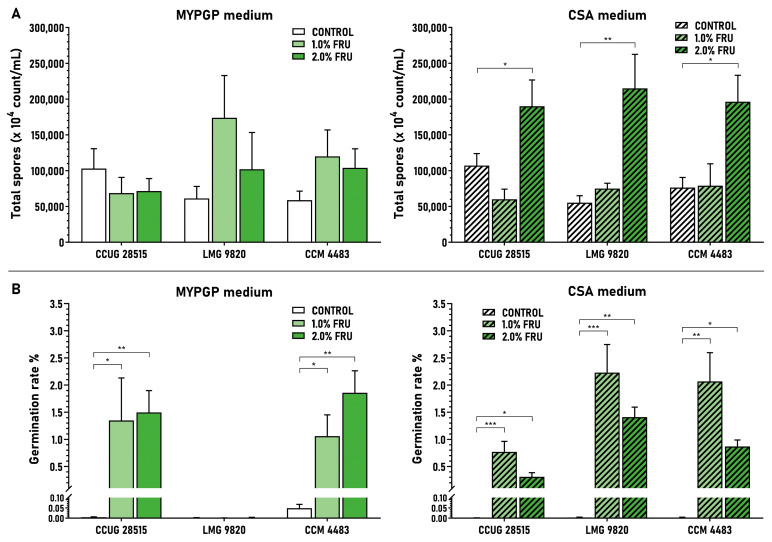
Effect of fructose on total spore production (**A**) and spore germination (**B**) of *P. larvae* strains with an ERIC I genotype. Data are expressed as the mean ± SEM. Data showing statistical significance are marked (* *p* < 0.05, ** *p* < 0.01, *** *p* < 0.001). Individual data sets represent results from 4–6 spore cultivation replicates. Spore germination rates were determined as the percentage of viable spores (grown on MYPGP plates supplemented with germination factors) to total spores (microscopically determined) present in the spore cultures.

**Figure 6:**
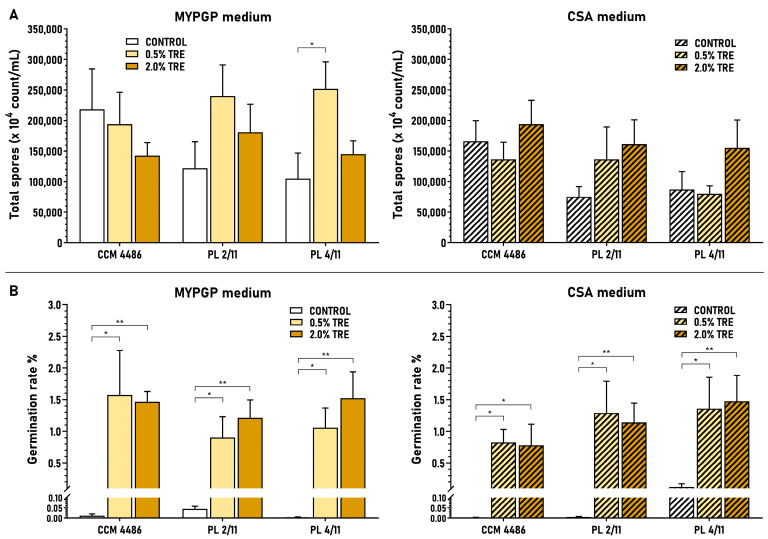
Effect of trehalose on total spore production (**A**) and spore germination (**B**) of *P. larvae* strains with an ERIC II genotype. Data are expressed as the mean ± SEM. Data showing statistical significance are marked (* *p* < 0.05, ** *p* < 0.01). Individual data sets represent results from 4–5 spore cultivation replicates. Spore germination rates were determined as the percentage of viable spores (grown on MYPGP plates supplemented with germination factors) to total spores (microscopically determined) present in the spore cultures.

Both saccharides showed a mostly positive effect on the total production of spores (viable and inviable), with variations existing between strains, saccharide concentrations and agar media. Fructose showed positive effects in almost all cases of ERIC I strains on both agar media except for one strain (CCUG 28515) at 1% and 2% concentrations on MYPGP plates and 1% concentration on CSA plates (Figure 5A). Trehalose showed positive effects in two ERIC II strains at 0.5% and 2% concentrations on MYPGP plates and in all strains at 2% concentration on CSA plates (Figure 6A). Generally, the saccharides, besides those several cases, did not affect the total spore production of the strains extensively; the quantitative ratios calculated between the average concentration of spores gained on saccharide media versus control basal media were 0.7–2.8-fold for fructose on MYPGP plates and 0.6–3.9-fold on CSA plates, and 0.7–2.4-fold for trehalose on MYPGP plates and 0.8–2.2-fold on CSA plates.

On the other hand, the saccharides vastly enhanced the germination ability of the spores of all corresponding strains at all concentrations tested, except the very poorly sporulating strain LMG 9820 on MYPGP plates. The average germination rates achieved on saccharide media were increased in comparison to the control basal media from values lower than 0.05% to 0.31–2.23% in the ERIC I strains (Figure 5B) and from values lower than 0.12% to 0.78–1.57% in the ERIC II strains (Figure 6B). The average germination rates were improved 21.9–314-fold on MYPGP plates and 215–711-fold on CSA plates in the first case, and 19.9–400-fold on MYPGP plates and 11.1–358-fold on CSA plates in the second case.

## 4. Discussion

Several procedures have been developed for in vitro preparation of spores from different *P. larvae* strains with the aim to reach efficient sporulation at different *P. larvae* strains. The basic procedure, by which satisfactory sporulation of many strains was achieved, uses sodium pyruvate (0.1%) and glucose (0.2%) as factors supporting sporulation on MYPGP agar plates [52]. This procedure was included among the “Standard methods for AFB research” [54]. The other procedure that has been used successfully for spore preparation from a number of *P. larvae* field isolates employs CSA agar slants [39]. The third procedure developed, named the UNLV method [55], utilizes TSA plates. It provided higher spore yields compared to the standard protocol of de Graaf et al. [54] in a test where three *P. larvae* strains were evaluated [59]. The UNLV method differs from the other protocols in several factors, such as plate inoculation, with very high numbers of vegetative cells, cultivation of plates in an incubator with 5% CO_2_ atmosphere and purification of the prepared spores through a 20–50% HistoDenz gradient. Moreover, a method employing the liquid medium TMYPG was developed for sporulation [53] but it turned out that a lot of *P. larvae* strains sporulate poorly in liquid medium [54]. It is not clear which of these procedures is the most suitable for efficient sporulation of the majority of *P. larvae* strains. It seems that none of them is universal—working satisfactorily with any arbitrary strain. One reason could be that the sporulation media used in the given protocols do not contain all the sporulation factors necessary for efficient sporulation of genetically diverse *P. larvae* strains. The data obtained in this study corroborate this assumption. 

The first and main result of this work was discovering new ERIC genotype-selective sporulation factors for the bacterium *P. larvae*. These sporulation factors are the individual saccharides fructose and trehalose added at higher concentrations to agar media. We demonstrated that fructose at 1–2% and trehalose at 0.5–2% concentrations in MYPGP or CSA agar media significantly enhance the average yields of the viable heat-resistant spores of the ERIC I and ERIC II strains, respectively, in comparison with the yields achieved on the basal agar media themselves. Both saccharides at a final concentration of 2% in the agar media were identified to be generally the most suitable for obtaining the highest or high average spore yields in most strains tested. The individual basal media employed for fructose/trehalose supplementation were found to affect spore yields partially and in a strain-specific manner. CSA–fructose/trehalose agars turned out to be more universal sporulation media than MYPGP–fructose/trehalose agars; all tested strains showed increased sporulation on them. On the other hand, the MYPGP–saccharide agars rendered higher spore yields than CSA–saccharides agars in some strains; therefore, they can be considered for certain strains as better media than the CSA–saccharide ones. It is necessary to note that one ERIC I strain (LMG 9820), which is often used as a reference strain in AFB research, displayed unexpected behaviour on the MYPGP–fructose media—very poor sporulation. It seems that the MYPGP basal medium is insufficient for sporulation of this strain. This is interesting in connection with our previous finding that this strain differs from the other ones also by its greater sensitivity to 10-HDA, an antibacterial royal jelly fatty acid [31]. These two observations indicate a singularity of this strain in some properties, the background of which is unknown and could be interesting to investigate. 

The second significant result of this work is the finding of the way in which higher fructose and trehalose concentrations in media enhance the production of viable spores of *P. larvae* strains. It was demonstrated that they massively improve the germination ability of the spores produced and, to a much lesser extent or not at all (in some strains, saccharide concentrations and agar media), the overall production of spores. At present, it is not clear which mechanisms participate in improvements of spore germination. We assume that they are associated with the spore maturation process, which has to take place to such an extent to form completely matured spores capable of germination in suitable conditions. This concept, together with our findings, corresponds well with existing knowledge about the sporulation of various *Bacillus* spp. Multiple sporulation studies on these bacteria have demonstrated that the composition of the sporulation medium has a strong impact on the structure and composition of their spores and on the speed and rate of spore germination. Moreover, studies have revealed that the composition of the medium also has an impact on the spore properties, such as resistance to heat, UV light and chemical agents (reviewed in [65]). The last of these is of great importance. It is probable that the medium composition also plays an important role in determining the properties of the prepared *P. larvae* spores. It prompts us to speculate that the medium composition could also affect the spore properties associated with *P. larvae* pathobiology in bee larvae [5]; for example, the sensitivity of the germinating spores to antibacterial substances in the larval diet in bioassay investigations. Based on this consideration, we believe that the use of fructose and trehalose in sporulation media is significant not only for the preparation of larger amounts of viable spores from the *P. larvae* ERIC I and ERIC II strains but also for preparation of spores with properties corresponding to those of native spores produced in diseased larvae. The use of such spores in various in vitro or in vivo studies could offer greater certainty that the findings gained with them will correspond to the reality occurring in natural conditions in infected larvae and colonies.

The third important result of this work is the finding that other saccharides are also able to positively affect *P. larvae* sporulation: 0.5% maltose and 0.5% sucrose did in most strains tested, and 1% glucose did in three strains. The average improvements in spore yields obtained with these saccharides versus the controls were, however, lower and showed more variation among the tested strains and between agar media than was observed for 1% fructose and 0.5% trehalose. Nevertheless, the observed effects of these saccharides suggest their possible utilization in combinational application with fructose or trehalose. We suppose that some saccharide combinations could have higher sporulation efficiencies than fructose or trehalose alone. Further investigation is needed to find out whether such combinations actually exist.

The results obtained in this study resemble in many aspects the results of other authors investigating the effects of higher concentrations of different individual saccharides on the sporulation of other bacterial species. Uemura and Hamasaki [66] studied the effects of six saccharides on the sporulation of two food poisoning strains of *Clostridium perfringens* type A at 0.1–4% concentrations in two liquid media. Five of them (fructose, galactose, lactose, sucrose and raffinose) showed a positive effect on sporulation at certain strain–medium–saccharide–saccharide concentration combinations. The spore yields for some combinations were increased 1.1–888-fold versus the basal media. The higher spore yields were caused by the increased cell growth and/or enhanced sporulation rate of *C. perfringens*. Interestingly, they observed a negative effect of glucose on the sporulation of both *C. perfringens* strains at all concentrations in both media. In the case of *P. larvae*, we also observed a negative effect of 1% glucose in agar media but only in some strains. Mazmira et al. [67] examined the effect of 0.8% glucose, fructose, galactose, sucrose, lactose and maltose on the growth and sporulation of *Bacillus thuringiensis* MPK13 in a liquid medium. The individual saccharides increased the spore yields 6–33-fold versus the values reached in the basal medium. Glucose, sucrose and maltose had the biggest effect on sporulation rate. The enhancements in spore yield with these saccharides were caused by similar processes to those we found for *P. larvae* sporulation. They varied for individual saccharides and included a higher cell growth (1.2–2.8-fold increases versus the control, except for galactose, which had no effect) and enhanced sporulation rate (5–11.7-fold increases). The contribution of an enhanced sporulation rate on the production of viable spores was mediated by an increased germination ability, but it was not so strong in *B. thuringiensis* compared to *P. larvae*. This could be associated with the fact that the production of viable *B. thuringiensis* spores, unlike the production of viable *P. larvae* spores, was already relatively high in the basal medium.

All five tested saccharides enhancing in vitro sporulation of *P. larvae* are natural constituents of honeybee larvae where they can occur in different amounts. They come from ingested components of larval food (royal jelly and honey) or are components of larval bodies [60,61,62,63,64,68]. The two most potent saccharides in sporulation, fructose and trehalose, probably also belong to the most abundant saccharide components of larvae. Fructose makes up 3–13% of royal jelly [60], which is the food produced by honeybees for 1–3-day-old larvae, and up to 32–44% of honey [61], which is an important food component of larvae older than 3 days. Trehalose is the major saccharide component of the larval body—it represents up to 65.8% of carbohydrates isolated from queen larvae younger than 5 days [68]. In lower amounts, it is also present in royal jelly (n.d. to 0.5%; [63]) and in honey (< 0.1–2.9%; [64]). The quantitative data suggest that both saccharides may be commonly present in high concentrations in larvae at the time of their killing by *P. larvae* and thus they naturally occur in the decaying larval mass in which the pathogen cells proliferate and sporulate. This may be the main evolutional and physiological reason why fructose and trehalose act at higher concentrations as significant sporulation factors of *P. larvae* in vitro in artificial agar media and very likely also in vivo in dead larvae. The question is why each of these saccharides exclusively supports sporulation of strains of only certain ERIC genotypes: ERIC I for fructose and ERIC II for trehalose. We assume that it could have two causes. The first is that the strains of these genotypes use these saccharides differently during cell proliferation and then later at the time of sporulation. The second is that those saccharides that are preferentially metabolized during cell proliferation are not employed or are less employed in sporulation metabolism. Our theory correlates with the following findings: (1) It has been demonstrated that *P. larvae* isolates of the ERIC I and ERIC II genotypes show differences in the metabolic profiles of growing cells [69]. All tested isolates with an ERIC I genotype (represented by Ab, ab and αβ genotypes) metabolized trehalose, some of them glucose and none of them fructose. On the other hand, all tested strains with an ERIC II genotype (AB genotype) metabolized fructose, most of them trehalose and some glucose during cell growth. (2) Fructose exclusively supported the sporulation of all tested ERIC I strains (strains not metabolizing it during cell growth) but none of the ERIC II strains (strains metabolizing it during cell growth) (finding presented here). (3) Glucose, the third highly abundant saccharide in royal jelly (4–8% content; [60]) and in honey (23–38% content; [61]) supported the sporulation of only some tested strains carrying both genotypes (findings presented here). This could be linked to the different metabolic profiles of glucose in growing and sporulating cells in the individual strains examined (not evaluated here). Further research is needed to confirm the proposed theory of selective and distinct usage of fructose, trehalose and glucose during growth and sporulation of strains with different ERIC I and ERIC II genotypes. It will also be necessary to find an explanation for the selective action of trehalose on sporulation of ERIC II strains in association with the fact that most of strains with this genotype were capable of metabolizing it during cell growth (mentioned in point (1) above).

In sporulation experiments, we were met with one recurring phenomenon. Considerable variation in the spore yields occurred within trials concerning the same experimental replicates (particular strain, saccharide, its concentration and agar medium) and controls (particular strain and basal agar medium). Up to a 2000-fold difference was observed between the lowest and highest values determined in some cases. We assume that the main cause of these variations lay in the cultivation of varying numbers (500–1500) of bacterial colonies on individual sporulation plates. We presume that different colony numbers on the plates partially, diversely and strain-specifically affected some parameters that determine the sporulation efficacy, such as cell growth, the size of the colonies and others unknown. The existence of such a colony plating effect indirectly supports the following facts. There is knowledge that the presence of too high a number of *P. larvae* colonies on a plate may reduce the sporulation efficiency as well as that the maximum sporulation obtained in relation to the colony numbers on a plate may vary between bacterial strains [54]; also, it has been reported that spore yields of *P. larvae* strains (three tested strains) obtained on MYPGP plates using low colony numbers (50–200) are higher than those using higher colony numbers (1000–5000). In one of the tested strains, even a 20,000-fold difference in spore yields was observed between low- and high-colony plates [59]. Taking all these facts into account, we think that they could have practical implications for spore preparation from strains with unsatisfactory sporulation on fructose or trehalose agar plates (if such strains are found). In such cases, optimization of the number of colonies allowed to grow and sporulate on agar plates could help to improve spore yields.

The last significant result and achievement of this work is the proposal of a novel procedure for the in vitro production of spores from *P. larvae* ERIC I and ERIC II strains, which was suggested on the basis of the knowledge gained in this study. The procedure includes a main/basic procedure and its possible variants that are gradually applied according to the results achieved for the strains actually used, in the following order: (1) Bacterial cells (amounts yielding about 500 CFU) are primarily cultivated on CSA agar containing 2% fructose for the ERIC I genotype strains and 2% trehalose for the ERIC II genotype strains for 10 days. (2) If a strain gives an unsatisfactory spore yield under these conditions, its cell cultivation is performed on MYPGP agar with 2% of the corresponding saccharide. (3) If that also fails, more appropriate cultivation conditions are sought; firstly, the optimal saccharide concentration in the medium that provided the better yield in Steps (1) or (2), and secondly an optimal number of colonies for cultivation on agar plates. Moreover, we assume that there is also another factor whose application could help increase the performance of the proposed procedure: the incubation of bacterial cells under partially anaerobic conditions in a 5% CO_2_ atmosphere at either 35 or 37 °C. Such incubations were not tested in this work. Nevertheless, we presume their possible beneficial effects on the basis of knowledge that *P. larvae* sporulation can be suppressed by oxygen toxicity [70] as well as on the basis that some successful in vitro preparations of spores from *P. larvae* strains have been performed in incubators with 5% CO_2_ at 35 °C [58] and 37 °C [55,59]. Further, we assume that the usage of a 5% CO_2_ atmosphere may be also beneficial for determining the germination ability of spores (not applied here). Germination under 5% CO_2_ should be higher than in air. This was observed for germination of native spores harvested from an AFB-diseased brood on different agar media (including the MYPGP agar used here) [71]. Finally, we think that for preparation of quality stocks of viable *P. larvae* spores it is necessary to centrifuge spores in a HistoDenz or Nycodenz gradient as the last purification step [31,55,59]. Centrifugation enables both a significant increase in the proportion of viable spores in culture, by removing the bulk of the unviable spores from it, as well as cleaning it of cell debris. Thus, we strongly recommend using this step in the spore preparation procedure suggested above.

## 5. Conclusions

This study demonstrated that two saccharides, fructose and trehalose, representing the quantitatively abundant components of honeybee larvae, significantly support the production of viable *P. larvae* spores when present at higher concentrations in the tested agar media. The knowledge gained about sporulation of the ERIC I and ERIC II strains under these conditions has been transformed into a proposal for a novel procedure for *P. larvae* spore preparation in vitro, which has the potential to produce spores with similar or the same properties as have the spores produced under natural conditions in diseased larvae. We suppose that the suggested procedure could be beneficial for preparing spores from diverse *P. larvae* strains for different studies concerning AFB. We also believe that employing the procedure with other *P. larvae* reference strains and field isolates will overcome a partial limitation of this study connected with the restricted number of strains examined and will validate its general quantitative and qualitative performance. In addition, some matters concerning the role of saccharides and other factors in in vitro and in vivo *P. larvae* sporulation were not explained here and further research is needed to clarify them.

## Data Availability

No other supporting data exist.
